# Development of a high-throughput assay to measure measles neutralizing antibodies

**DOI:** 10.1371/journal.pone.0220780

**Published:** 2019-08-15

**Authors:** Esmeralda Alvarado-Facundo, Susette Audet, William J. Moss, Judy A. Beeler

**Affiliations:** 1 Division of Viral Products, Office of Vaccines Research and Review, Center for Biologics Evaluation Research, Food and Drug Administration, Silver Spring, Maryland, United States of America; 2 International Vaccine Access Center, Bloomberg School of Public Health, Johns Hopkins University, Baltimore, Maryland, United States of America; Institut Pasteur of Shanghai Chinese Academy of Sciences, CHINA

## Abstract

Measles virus is highly infectious and remains a leading cause of vaccine preventable deaths in children. Neutralizing antibody responses elicited by measles virus infection or immunization are a serological correlate of protection. We describe a high-throughput neutralization assay to improve surveillance for measles immunity. Measles virus-antibody mixtures were incubated on Vero cell monolayers and 24 hours later cell-lysates harvested and subjected to one-step SYBR green RT-qPCR to amplify a target sequence within the measles virus nucleoprotein gene. Neutralization endpoint titers were interpolated to determine the dilution that inhibited the relative amplicon copy number by at least 90% compared to the mean signal obtained in virus control wells in the absence of serum. Anti-measles virus and anti-measles hemagglutinin antisera specifically neutralized measles virus in the microneutralization RT-qPCR assay while pre-immune sera and sera raised against other viruses did not. The microneutralization RT-qPCR assay obeyed the Percentage Law for measles virus inputs ranging from 100–5000 TCID_50_/well. The linear range of the assay corresponds to measles antibody concentrations of 30 to 3000 mIU/mL. Bland-Altman analysis and two-way analysis of variance demonstrated that results obtained using the microneutralization RT-qPCR assay were comparable to those obtained using a plaque reduction neutralization test and correctly identified human serum samples that were seropositive (95% and 100%, sensitivity and specificity, respectively). Furthermore, these comparisons suggest that a concentration of 300 mIU/mL may be a conservative cut-point to use to identify individuals likely to be protected against severe measles disease when the endpoint is based on 90% inhibition of virus replication. Measles virus microneutralization RT-qPCR is a rapid, sensitive, specific, and robust assay for detecting measles neutralizing antibodies that may help to improve immunization strategies nationally and achieve measles elimination globally.

## Introduction

Measles virus (MV) is highly infectious and remains a leading cause of vaccine preventable deaths in children. Neutralizing antibody responses elicited by MV infection or immunization are a proven serological correlate of protection, with concentrations of measles antibodies ≥200 mIU/mL protecting against severe disease [1, 2]. A rapid, high-throughput assay to quantitate levels of measles neutralizing antibodies can provide information about individual immunity, improve estimates of herd immunity, and identify measles-susceptible individuals for immunization. This information will enable countries to target immunization programs for regional measles elimination and facilitate global measles eradication.

Prior to the development of a vaccine, MV infected almost all children, causing more than one million deaths per year. An effective live-attenuated measles vaccine (MVac) became available in 1963, and subsequent international efforts to increase vaccination rates resulted in an 80% decrease in global measles mortality between 2000 and 2017 [3]. However, immunization rates continue to fall short in many regions, with population immunity below the 93–97% needed to interrupt transmission. In the absence of high levels of population immunity, measles outbreaks occur, and endemic circulation of MV continues in some countries.

Measles elimination is an important part of the WHO Global Vaccine Action Plan. The current goal is to eliminate measles in five WHO regions by 2020 [4]. Data from measles seroprevalence studies support and verify estimates of MVac uptake in settings with minimal transmission of wild-type MV. In addition, serosurveys can identify susceptible populations, assess persistence of vaccine-induced antibodies, help to monitor vaccine immunogenicity when measles-containing-vaccines are given in the context of a new formulation or schedule, and help optimize the age for MVac administration.

Enzyme-linked immunosorbent (ELISA) and plaque reduction neutralization (PRN) assays are traditionally used to assess seroprevalence of measles antibodies. ELISA kits are easy to use and provide results quickly. Nevertheless, these kits are designed to have a low, false-positive rate and relatively high levels of antibodies are needed for a positive result. Therefore, samples with low PRN measles antibody concentrations often give negative results when tested using ELISA. Consequently, ELISAs may underestimate measles immunity. In contrast, the PRN assay is sensitive and specific in identifying individuals protected against measles, but it is laborious, and the technology is not easily transferrable.

We describe a high-throughput, rapid, anti-measles virus IgG microneutralization assay with a RT-qPCR endpoint (MN-RT-qPCR) to improve the ability to screen and measure large numbers of serum samples for measles neutralizing antibodies.

## Material and methods

### Cells, viruses, and antibodies

Vero cells (ATCC CCL-81) were maintained in EMEM (Corning) as previously described [5]. Viruses used for rabbit immunizations included: low-passage wild-type Edmonston MV (provided by Dr. Paul Albrecht, FDA), Enders-Edmonston (Moraten) MVac (Merck), respiratory syncytial virus (RSV) strains A2 and B1 (provided by Drs, Chanock, Murphy, and Rubin, NIH) and human metapneumovirus (hMPV, provided by Dr. Buchholz, NIH). All were grown in Vero cells, purified, and UV-inactivated as previously described [5].

Monospecific rabbit polyclonal sera against RSV were prepared by immunizing animals with UV-inactivated sucrose-gradient-purified RSV-A2 or -B1 under a protocol approved by CBER IACUC as previously described [5]. Anti-sera against Moraten MVac, UV-inactivated Moraten MVac, and UV-inactivated Edmonston wild-type MV were generated in a similar manner using 10^4^ TCID_50_ dose equivalents. Additional antisera tested were: anti-canarypox vectored measles-hemagglutinin protein, anti-canarypox vectored measles-fusion protein (provided by Dr. Taylor, Wadsworth Center), anti-rubella (Fitzgerald Industries International, #60-RG04), anti-mumps (BEI #NR-4019), and normal guinea pig, goat, and rabbit sera.

De-identified, archived human serum samples from volunteers were obtained with informed consent and tested with approval from the US Food and Drug Administration Research Involving Human Subjects Committee (RIHSC #03-154B). These samples represent pre- and post-vaccination sera having a PRN ND_50_ values of <4 to 700 mIU/mL. Samples were selected because they had sufficient volume for testing and no obvious evidence of hemolysis.

The WHO 3rd International Standard (IS) 97/648 (WHO 3 IS, NIBSC) was reconstituted per instructions yielding assigned units of 3000 mIU/mL, and a portion further diluted 1:10 in DPBS. Human immune globulin to RSV (huIgG, 10% IgG, BEI #21973) was diluted to 1% IgG concentration in DPBS [6]. Human sera depleted of IgG, IgM, and IgA was obtained from SunnyLab (UK).

All sera were heat-inactivated for 30 minutes at 56°C and stored at -20°C prior to use.

### Measles RNA standard

Measles RNA standard was obtained by infecting Vero monolayers with low-passage Edmonston MV at 0.1 MOI for 48 hours. Infected cells were harvested, pelleted at 500 *x g* for 5 minutes, and total RNA extracted using the RNeasy Mini Kit (Qiagen) according to the manufacturer’s instructions. The quality and concentration of the RNA were determined by spectrophotometry.

### MN-RT-qPCR assay for MV

The MN-RT-qPCR assay was conducted as previously described [5]. Briefly, serial dilutions of antibody or serum were mixed with low-passage Edmonston MV and incubated at 37°C for 2 hours. Afterwards, 100 μL of virus/antibody mixtures were transferred to Vero cell monolayers in duplicate. Samples were run in parallel with the WHO 3 IS. At 24 hours post-infection, cell monolayers were washed twice with DBPS and 100 μL iScript Sample Preparation Reagent (SPR, Bio-Rad) was added to monolayers, incubated 3 minutes at room temperature, and cell lysates transferred well-by-well into a clean 96-well U-bottom plate (SPR lysates) and assessed by RT-qPCR as described below or stored at -20°C until use.

Three primer pairs targeting conserved regions of the MV-nucleoprotein (N) gene were synthesized and assessed in the RT-qPCR assays: N3-F/N3-R, NPB1/NPB2, and N-F/N-R [7–9]. To optimize each primer pair, a temperature gradient was used to determine the optimal annealing temperature and concentrations ranging from 100/100 nM to 1000/1000 nM were tested to determine the optimal primer concentration. The following parameters were evaluated: lowest threshold cycle (C_t_) for detection, the ability to detect at least 10^2^ copies of the RNA target, an amplification efficiency between 90–120%, and a R^2^ value exceeding 0.99 over 5–6 log_10_ range of input RNA. Based on the performance of each set, the NPB1/NPB2 primer pair had the highest amplification efficiency and was used in all subsequent experiments.

After optimization, RT-qPCR reactions contained: 400 nM of primers, 1X iTaq Universal SYBR Green 1-Step Reaction Mix (Bio-Rad), 1 μL sample (SPR lysate), and water to 10 μL. RT-qPCR was performed in a CFX-96 instrument (Bio-Rad) using the following conditions: 50°C for 10 minutes (1 cycle)/ 95°C for 1 minute (1 cycle)/ 95°C for 10 seconds, 67°C for 30 seconds (40 cycles) followed by generation of disassociation curves for each sample. In each RT-qPCR run, the following were included: RNA standard (diluted using 6, 10-fold serial dilutions in cell lysates from uninfected Vero cells, corresponding to 10 ng/μL to 0.0001 ng/μL RNA), no reverse transcription (RT) controls, and no template controls. The neutralization titers (ND_90_) were calculated as follows: 1) RNA standard was assigned arbitrary units of 10^6^ to 10 copy numbers and this standard was used to verify the performance of each PCR run and plot a standard curve to determine the relative gene copy number; data are automatically accrued into Excel for endpoint determination; 2) at each dilution, ratios of relative copy numbers were determined by comparing the number of wells containing virus/antibody mixtures to the mean value obtained from virus-infected controls wells in the absence of serum; 3) neutralization titers were estimated by plotting the slope of the ratios immediately above and below the dilution associated with 90% inhibition of relative amplicon copy numbers using the following formula: ND_90_ titer = dilution immediately above 90% × ((y+b) ÷ m) where y = 0.1. 4) titers were standardized against the WHO 3 IS according to the formula: [(3000 mIU/mL assigned unitage for WHO 3 IS ÷ WHO 3 IS titer) × titer of test sample] and reported as a concentration of measles antibody in mIU/mL [10]. Samples with no detectable measles neutralization in the first dilution were assigned a value of 4 for purposes of calculating geometric mean titers (GMTs), which was equivalent to a concentration of 32 mIU/mL.

### Acceptance criteria for MN-RT-qPCR assay

The MN-RT-qPCR assay was considered valid if the following criteria were met: 1) cell control monolayers were intact by microscopic inspection; 2) infectious titer of the input MV was 500–5000 TCID_50_/well; 3) titers of the replicates were within 3-fold of each other; 4) titer of WHO 3 IS within the GMT of 490 ± 2SD (range = 294–686); 5) titer of the huIgG (BEI NR #21793) was within the historical GMT of 297 ± 2SD (range = 147–447); 6) no inhibition of the PCR signal in the negative control; 7) mean Ct value for virus control was ≤24; 8) RT-qPCR efficiency was 90–120%; 9) RNA standard curve had a R^2^ ≥0.980; and 10) no RT control, no template control, and cell controls all had Ct values ≥34. The GMTs for WHO 3 IS and huIgG (BEI NR# 21973) were initially set based on 8 replicates tested in 4 valid assays.

### Edmonston MV kinetics by RT-qPCR

The ability of RT-qPCR to monitor replication of MV in Vero cells over time was assessed to determine the earliest time-point with a reliable PCR signal for detecting MV. Briefly, Vero monolayers were seeded into 96-well plates and the following day infected with 500 TCID_50_/well of MV while control wells received medium only. Plates were then incubated for 1 hour at 37°C, 5% CO_2_ followed by removal of the inoculum, washed twice with DPBS, and re-fed with OptiPro with glutamine. Cell lysates were harvested at time 0 and every 24 hours thereafter for 4 days and assessed by RT-qPCR as described above.

### Compliance with Percentage Law

According to the Percentage Law, antibody titers will not vary as the quantity of virus changes as long as antibodies are in molar excess [11, 12]. To determine if the MN-RT-qPCR assay complies with the Percentage Law, huIgG (BEI NR #21793) was diluted to concentrations of 1%, 0.5%, and 0.01% IgG to simulate samples with high, medium, and low measles antibodies [expected PRN 50% neutralization (ND_50_) results of 2500, 250, and 25 mIU/mL, respectively] and tested against MV inputs of 100, 500, 1000, 2500, and 5000 TCID_50_/well. The assay was harvested 24 hours post-infection and neutralization endpoints assessed using MN-RT-qPCR as described above.

### Linear range of the MN-RT-qPCR assay

Linearity was assessed by testing 10 independent dilutions of WHO 3 IS over a wide range of measles antibody concentrations. WHO 3 IS was diluted in serum-free EMEM to represent expected measles antibody concentrations of 750, 375, 188, 94, 47, 23, 12, 6, 3, and 1.5 mIU/mL. Each diluted sample was then tested using 10 2-fold serial dilutions in duplicate and tested in two independent assays as described above. Titration curves were plotted in Prism (GraphPad Software) using the log_10_ of the 90% endpoint inhibitory titer for each dilution versus the log_10_ of the dilution. A correlation coefficient was calculated.

### MV PRN assay

The PRN assay was performed as previously described [13]. All samples were tested in parallel with the WHO 3 IS and ND_50_ endpoint titers calculated using the Kärber method [10]. In some cases, to facilitate comparisons across assays, raw data were re-analyzed to obtain ND_90_ results and standardized against the WHO IS [10]. In the PRN assay, samples with no detectable neutralization at the first dilution were assigned a titer of 2 for the purposes of calculating GMTs. Based on the performance of the WHO 3 IS, a ND_50_ value of 2 is equivalent to a ND_50_ value of 4 mIU/mL and a ND_90_ value of 2 is equivalent to a concentration of 60 mIU/mL.

### Statistics

ND_90_ results were estimated by linear interpolation in Excel. Linearity, ROC, Bland-Altman, and correlation analyses were conducted using Prism. The measles antibody GMCs from PRN and MN-RT-qPCR assays were compared using the Mann-Whitney test (Instat GraphPad).

## Results

### Optimization of MN-RT-qPCR assay

**Fig 1:**
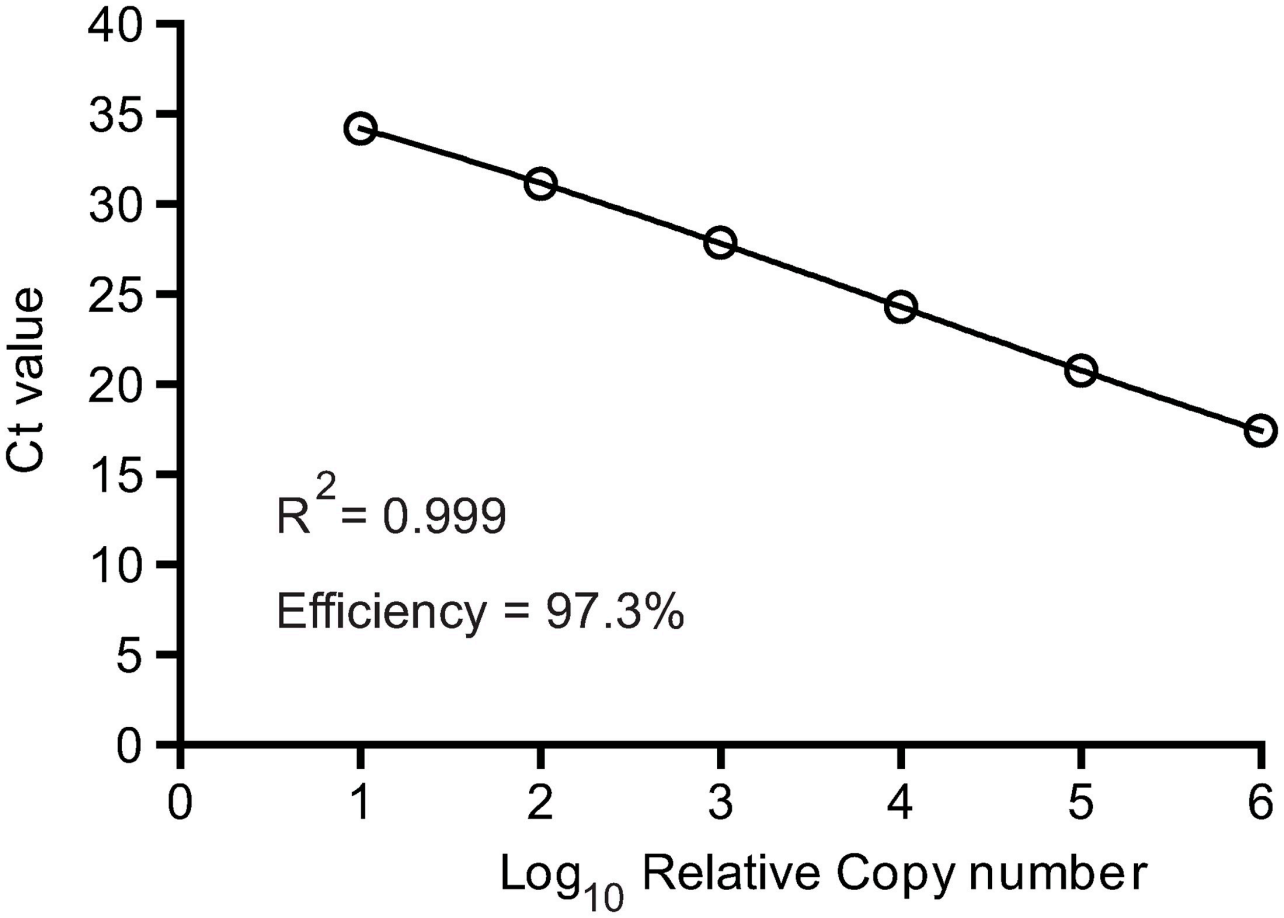
SYBR Green RT-qPCR targeting the N gene of MV. Total RNA was purified from Vero cells infected with low-passage Edmonston MV and serially diluted (10-fold) into Vero cell lysate obtained from uninfected Vero cells (10 ng/μL to 0.0001 ng/μL). One microliter of each dilution was subjected to one-step SYBR green RT-qPCR. The mean threshold cycle (Ct, N = 2) is plotted against log_10_ for each RNA standard dilution.

Three MV-N primer sets NPB1/NPB2, N-F/N-R and N3-F/N3-R were identified and used to amplify MV-N gene targets using serial 10-fold dilutions of MV RNA spiked into Vero cell lysates (Table 1). The optimal annealing temperatures and concentrations for both forward and reverse primers were 67°C and 400 nM, 57°C and 400 nM, and 60°C and 600 nM for NPB1/NPB2, N-F/N-R, and N3-F/N3-R, respectively (S1 and S2 Figs). All primers performed well but NPB1/NPB2 had a higher efficiency in amplifying the MV-N gene target than the other primer pairs (PCR efficiency of 97.3% and R^2^ of 0.99) and therefore this primer set was used for all subsequent experiments (Fig 1).

**Table 1:** Primer pairs targeting the N gene of measles virus.

Primer	Sequence	Amplicon size
NPB1NPB2	GATCCGCAGGACAGTCGAAGGTCAGGGTAGGCGGATGTTGTTCT	169
N-FN-R	AGTGAGAATGAGCTACCGTGTCTAGGGGTGTGCC	151
N3-FN3-R	TGGCATCTGAACTCGGTATCACTGTCCTCAGTAGTATGCATTGCAA	75

To optimize the incubation time, virus/antibody mixtures were incubated for 1 or 2 hours prior to transferring mixtures to Vero monolayers. Measles antibody concentrations were within 2-fold of each other irrespective of the incubation time. Measles antibody concentrations obtained following a 2-hour incubation were numerically higher than those obtained after a 1-hour incubation, albeit the difference was not significant (S1 Table).

To estimate non-specific inhibitory effects of serum in the MN-RT-qPCR assay, immune globulin-depleted human sera was tested in the assay as previously described [5]. No inhibition of the PCR signal was observed over a broad range (1:2 to 1:128; S3 Fig). Based on these observations, all samples were tested using a starting dilution of 1:8 to accommodate low sample volumes.

### Replication kinetics of Edmonston MV by RT-qPCR

**Fig 2:**
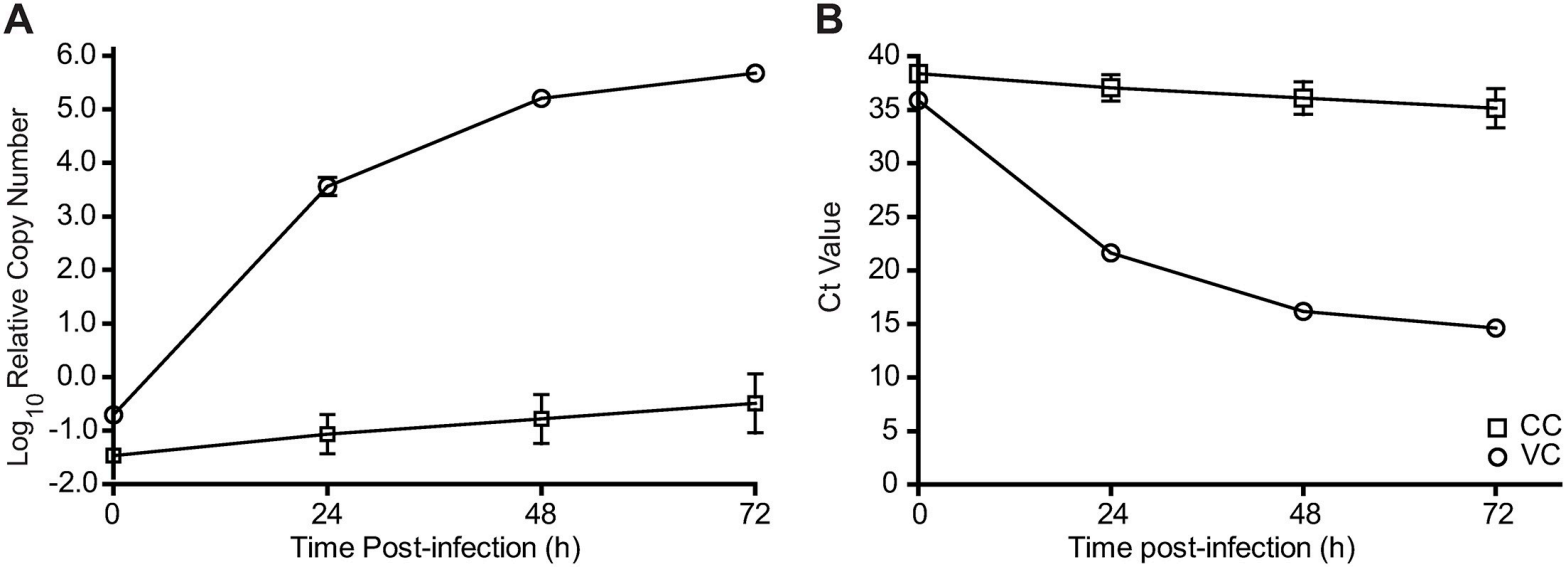
Replication kinetics of MV. Vero cells were seeded in 96-well plate. The next day, monolayers were infected with 500 TCID_50_ per well of low-passage Edmonston MV or mock infected with media only. The procedure followed the standard MN-RT-qPCR protocol except in absence of antibodies. At the indicated times, cell lysates were prepared using Bio-Rad SPR and subjected to one-step SYBR green RT-qPCR. Each point represents the mean of 8 replicates of measles-infected Vero cells (◯) or uninfected Vero cells (☐) for (A) relative RNA copy numbers and (B) Ct value.

The ability of RT-qPCR to detect replication of low-passage Edmonston MV in Vero monolayers was assessed over time to determine the earliest time-point producing a reliable MV-N gene PCR signal. The MV-N amplicon in measles-infected cell lysates increased with time (Fig 2A), with a corresponding decrease in Ct value over 72 hours while the signal from uninfected cells was unchanged (Fig 2B). Since amplification of MV-N gene was significantly above background as early as 24 hours post-infection, this time-point was chosen for further study.

### Specificity of the MN-RT-qPCR assay

Specificity was assessed by measuring the ability of mono-specific polyclonal antisera to neutralize MV (Table 2). Rabbit anti-UV-inactivated-Edmonston wild-type MV, anti-Moraten, and anti-UV-inactivated-Moraten antisera neutralized MV efficiently with geometric mean concentrations (GMCs) of 397, 3956, and >6349 mIU/mL, respectively, while normal rabbit serum was negative. Antisera to canarypox measles (CPMV)-hemagglutinin protein (HA) had a neutralizing antibody concentration of 1693 mIU/mL whereas anti-CPMV-fusion protein (F) did not neutralize MV in this assay. In contrast, anti-CPMV-F antisera neutralized MV albeit at low concentrations in the PRN assay (S2 Table). Anti-mumps, anti-rubella, normal guinea pig, and normal goat sera did not neutralize MV as expected. Likewise, rabbit anti-UV-inactivated-RSV-A2, anti-UV-inactivated-RSV-B1, and anti-UV-inactivated-hMPV did not neutralize MV but were able to effectively neutralize the homologous viruses (S2 Table).

**Table 2:** Specificity of MN-RT-qPCR assay using polyclonal antisera.

Sample	GMC (mIU/mL)
Normal rabbit serum	≤32
Normal guinea pig serum	≤32
Normal goat serum	≤32
Rabbit anti-UV-inactivated-RSV A2	≤32
Rabbit anti-UV-inactivated-RSV B1	≤32
Rabbit anti-UV-inactivated-hMPV	≤32
Guinea pig anti-Mumps virus, Enders	≤32
Goat anti-Rubella, HPV77	≤32
Rabbit anti-Canarypox Measles-F	≤32
Rabbit anti-Canarypox Measles-HA	1693
Rabbit anti-UV-inactivated-Edmonston measles wild-type	397
Rabbit anti-Moraten measles vaccine virus	3956
Rabbit anti-UV-inactivated-Moraten measles vaccine virus	>6349

### Compliance with Percentage Law

**Fig 3:**
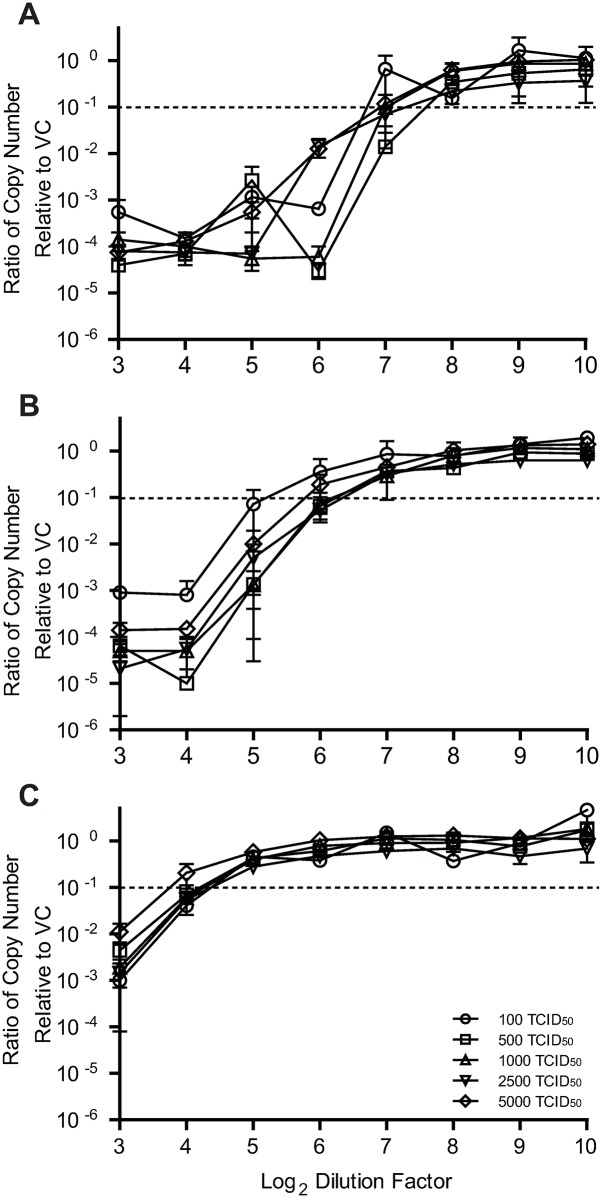
Compliance with Percentage Law. Vero cells were seeded in 96-well plates. The next day, 2-fold serial dilution series of huIgG (BEI NR #21793) were prepared starting at an initial IgG concentration of 1%, 0.5%, or 0.1%, representing high, medium, or low MV neutralizing antibodies, respectively. An equal volume of low-passage Edmonston MV diluted to 100, 500, 1000, 2500, or 5000 TCID_50_/well was mixed with the sample and incubated at 37 °C for 2 hours. After incubation, mixtures were transferred to Vero monolayers and 24 hours post-infection, cell lysates were prepared using Bio-Rad SPR and subjected to one-step SYBR green RT-qPCR. RNA copy numbers for wells exposed to virus-antibody mixtures were normalized to the mean value obtained from virus-infected control wells in the absence of immunoglobulin. Each point represents the mean of 2 replicates for: (A) huIgG at 1%, (B) huIgG at 0.5% or (C) huIgG at 0.1%.

Neutralizing activity of huIgG (BEI NR #21793) diluted to concentrations of 1%, 0.5%, and 0.1% IgG was measured over a wide range of virus inputs (100 to 5000 TCID_50_/well) (Fig 3). ND_90_ results for huIgG varied less than 2.5-fold as the quantity of input virus increased irrespective of the starting IgG concentration. ND_90_ results for the huIgG at 1% IgG were 190, 153, 127, 171, and 118 (Fig 3A), for huIgG at 0.5% IgG were 32, 68, 67, 76, and 54 (Fig 3B), and for huIgG at 0.1% IgG (0.1 mg/mL IgG) were 18, 17, 18, 19, and 13 (Fig 3C) for virus inputs of 100, 500, 1000, 2500, and 5000 TCID_50_, respectively. The sample with the lowest measles antibody titer, huIgG at 0.1% IgG, had the least variability across the virus inputs providing assurance that the MN-RT-qPCR assay could reliably identify serum samples with low concentrations of measles neutralizing antibodies.

### Linearity of MN-RT-qPCR

**Fig 4:**
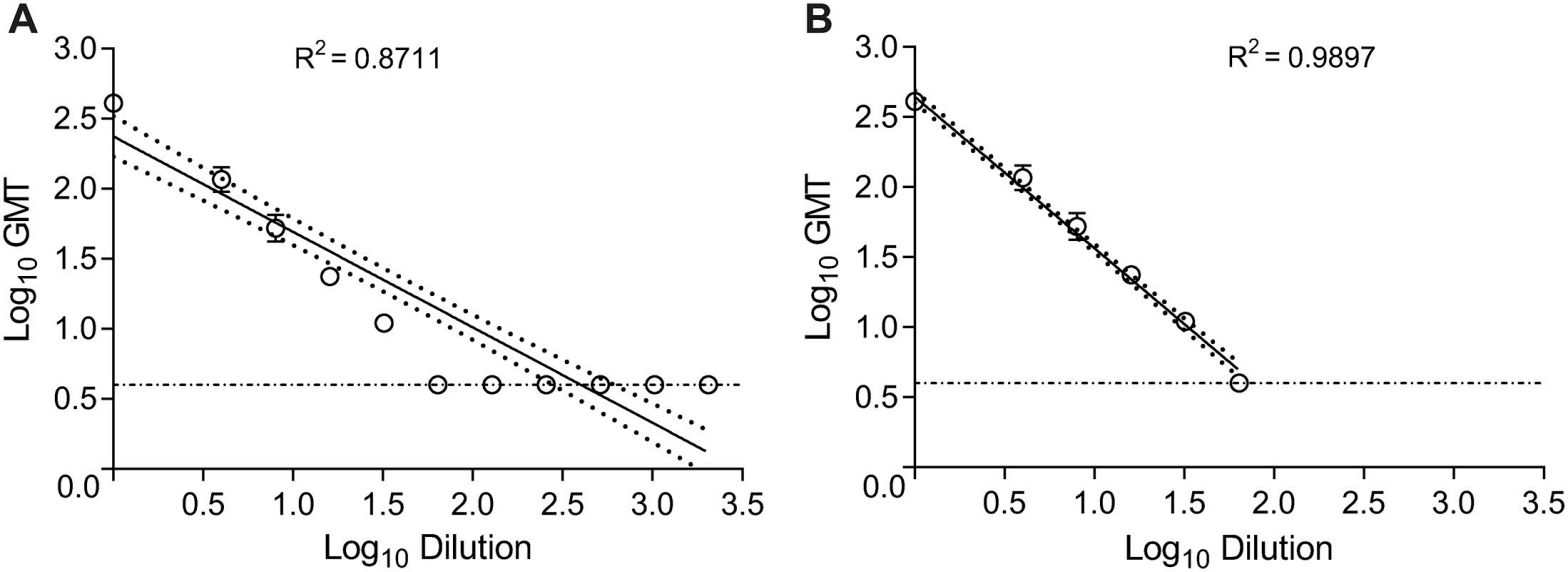
Linearity of MN-RT-qPCR assay. The linearity of MN-RT-qPCR assay was assessed by testing 10 samples with varying measles antibody concentration. All samples were tested in duplicate in two independent assays. The GMT titers were plotted using the log of the GMT for each sample versus the log of the reciprocal dilution and a correlation coefficient calculated. Each symbol represents the GMT ± standard deviation for each sample (N = 4). The horizontal dashed line represents the limit of detection of log_10_0.60 (titer of 4). The best fit line and 95% confidence bands are represented by the solid and dotted lines, respectively. The GMT for the undiluted sample was based on a back-calculation of all the diluted sample.

The linearity of the MN-RT-qPCR assay was determined by testing the ability of independent dilutions with varying measles antibody concentrations of the WHO 3 IS to inhibit MV. The log_10_ GMT for each sample was then plotted versus the reciprocal of the log_10_ dilution. This titration curve demonstrated that the MN-RT-qPCR assay was linear over a wide range of measles antibody titers with a correlation coefficient of R^2^ > 0.87 (Fig 4A). When the titer range was limited to 4 to 400, corresponding 30 to 3000 mIU/mL, the R^2^ > 0.98 (Fig 4B). The undiluted WHO 3 IS had a GMT of 400 which was within the historical GMT of 474 ± 100 when tested in 8 assays corresponding to a total of 48 determinants.

### Endpoints obtained by MN-RT-qPCR vs. PRN assays

**Fig 5:**
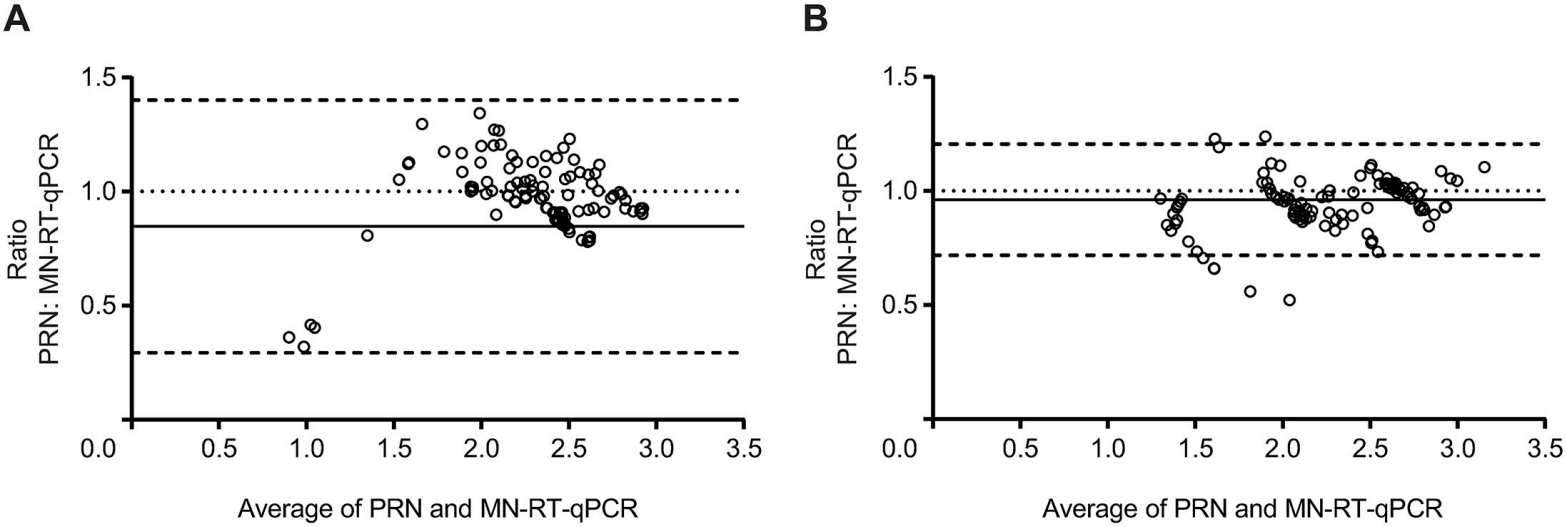
Bland-Altman plots of PRN vs. MN-RT-qPCR assays. Neutralization assays were performed with low-passage Edmonston MV using a panel of human serum samples (N = 136). The concentrations were log transformed prior to the analysis. The ratios of PRN to MN-RT-qPCR were plotted against the average of PRN and MN-RT-qPCR. (A) Analysis of PRN ND_50_ vs. MN-RT-qPCR ND_90_ values. (B) Analysis of PRN ND_90_ vs. MN-RT-qPCR ND_90_ values of measles neutralizing antibodies in milli-International units. The lines of no bias (dotted line at y = 1), mean bias (solid line), and lower and upper limits of agreements (dashed lines) are shown.

A total of 136 incurred human serum samples previously tested using the PRN assay (PRN ND_50_ values ranged from <4 to 700 mIU/mL) were tested in the MN-RT-qPCR assay. ND_90_ results were calculated and converted to a concentration of measles antibodies based on the performance of WHO 3 IS tested in parallel. The PRN ND_50_, PRN ND_90_, and MN-RT-qPCR ND_90_ GMCs for the human serum samples overall were 76, 108, and 133 mIU/mL, respectively. There were no significant differences between the antibody concentrations when the GMCs between the two assays were compared (PRN ND_50_ vs MN-RT-qPCR ND_90_ results p = 0.16 and PRN ND_90_ vs MN-RT-qPCR ND_90_ results p <0.06). GMCs obtained using individual incurred human serum samples were log transformed and analyzed for bias using both the Bland-Altman method and a correlation analysis using a scatterplot. These analyses showed that neutralization endpoints obtained using MN-RT-qPCR ND_90_ were in agreement with PRN ND_50_ results and had good correlation (Fig 5A and S4 Fig). The mean ratio discrepancy between the PRN ND_50_ and MN-RT-qPCR ND_90_ was 0.85, with lower and upper limits of agreement of 0.29 and to 1.4, respectively. To account for differences in neutralization endpoints (ND_50_ vs ND_90_), data derived from PRN assays were re-analyzed to calculate ND_90_ concentrations and then compared in a Bland Altman analysis with ND_90_ concentrations obtained using MN-RT-qPCR assay. Comparison of ND_90_ concentrations across the two assays yielded a mean bias of 0.96 and lower and upper limits of agreement of 0.72 and 1.2, respectively (Fig 5B).

### Estimation of concentration associated with protection against severe disease

**Fig 6:**
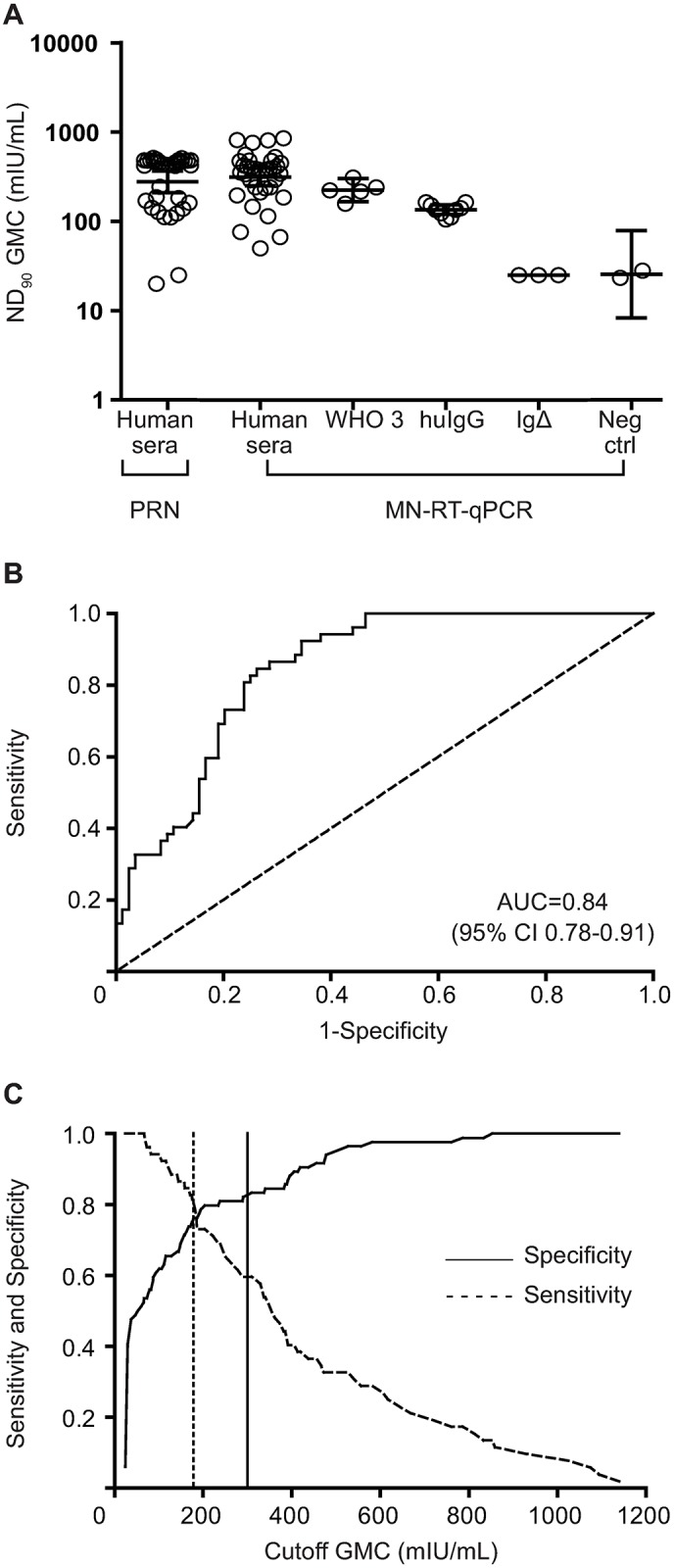
ROC analysis and estimation of concentration associated with protection in MN-RT-qPCR assay. (A) Plot of concentrations of human serum samples (N = 36) and control samples when tested in either PRN or MN-RT-PCR assays. The human serum samples were initially selected based on the ND_50_ PRN results of 180–220 mIU/mL, representing protective concentrations. WHO 3 IS and huIgG (BEI NR #21793) were specifically diluted to have an expected concentration of ~200 mIU/mL. The bars represent the 95% CI from the GMC (solid line) for each group. (B) ROC curve of concentrations of measles neutralizing antibodies as determined by MN-RT-PCR assay from individuals (N = 136) considered to be protective (>200 mIU/mL) or unprotected by traditional PRN assay. AUC represents the overall performance of the MN-RT-qPCR. An AUC of 0.5 (dash line) represents random guess. (C) The sensitivity (solid line) and specificity (dashed line) of the MN-RT-PCR assay plotted along the measles neutralizing antibody cut-off concentrations (mIU/mL). The dashed vertical line represents the theoretical protective concentration as determined by the Youden’s index. The solid vertical line represents the theoretical protective concentration as determined by empirical analysis of serum samples (see Fig 6C).

To empirically determine a protective threshold when using the MN-RT-qPCR assay, a subset of human sera samples (N = 36) with known PRN ND_50_ results of ~200 mIU/mL (range 180–220 mIU/mL) were tested using the MN-RT-qPCR method. The GMC of the 36 samples in the MN-RT-qPCR assay was ~300 mIU/mL (95% CI 251 to 395 mIU/mL), in close agreement with the calculated PRN ND_90_ value of 278 mIU/mL (95% CI 210 to 366 mIU/mL) for these samples (Fig 6A). Additionally, when the WHO 3 IS and huIgG (BEI NR #21793) were diluted to contain ~200 mIU/mL of measles antibodies prior to testing, these samples had ND_90_ values of 224 mIU/mL (95% CI 166 to 303) and 135 mIU/mL (95% CI 120 to 153 mIU/mL).

To further evaluate the ability of the MN-RT-qPCR assay to identify individuals with concentrations associated with protection (≥200 mIU/mL), a ROC analysis was performed using the GMC for 136 human serum samples. The accuracy with which the MN-RT-qPCR assay could identify protected individuals was good (area under the curve (AUC) = 0.84, 95% CI, 0.78 to 0.91) (Fig 6B). To determine an estimated concentration of measles antibodies needed for protection when using the MN-RT-qPCR assay, the sensitivity and specificity was plotted along varying cutoffs. The predicted concentration for protection was 180 mIU/mL with a Youden’s J index of 0.57 (Fig 6C).

### Sensitivity and specificity of the MN-RT-qPCR assay

Two-way comparisons were conducted using PRN and MN-RT-qPCR results (Table 3). When identifying measles seropositive (>4 mIU/mL for PRN and >32 mIU/mL for MN-RT-PCR assays) individuals, the MN-RT-qPCR had excellent agreement with ND_50_ results obtained using the PRN assay (96%, 131/136), with a sensitivity of 95% (96/101), specificity of 100% (35/35), positive predictive value (PPV) of 100%, and negative predictive value (NPV) of 88%. When identifying seroprotected individuals using the conventional cutoff of ≥200 mIU/mL in both assays, the agreement was 77% (105/136), sensitivity of 73% (38/52), specificity of 80% (67/84), PPV of 69%, and NPV of 83%. However, using a seroprotective threshold of ≥300 mIU/mL for the MN-RT-qPCR, the agreement was slightly lower at 74% (100/136), sensitivity of 60% (31/52), specificity of 83% (70/84), PPV of 69%, and NPV of 77%.

**Table 3:** Comparison of MN-RT-qPCR and PRN results.

Seropositive Threshold^a^							
MN-RT-qPCR cutoff (mIU/mL)	PRN cutoff(mIU/mL)		Sensitivity (%)	Specificity (%)	Accuracy (%)	PPV (%)	NPV (%)
	>4	≤4					
>32	96	0	95	100	96	100	88
≤32	5	35					
Seroprotective Threshold of 200 mIU/mL^b^							
MN-RT-qPCR cutoff (mIU/mL)	PRN cutoff(mIU/mL)		Sensitivity (%)	Specificity (%)	Accuracy (%)	PPV (%)	NPV (%)
	≥200	<200					
≥200	38	17	73	80	77	69	83
<200	14	67					
Proposed Seroprotective Threshold of 300 mIU/mL^c^							
MN-RT-qPCR cutoff (mIU/mL)	PRN cutoff(mIU/mL)		Sensitivity (%)	Specificity (%)	Accuracy (%)	PPV (%)	NPV (%)
	≥200	<200					
≥300	31	14	60	83	74	69	77
<300	21	70					

^a^A seropositive concentration is defined as PRN ND_50_ value of >4 mIU/mL and MN-RT-qPCR ND_90_ value of >32 mIU/mL.

^b^The conventional seroprotective concentration is defined as ≥200 mIU/mL, irrespective of assay used.

^c^The proposed seroprotective threshold of GMC of ≥300 mIU/mL was calculated on the testing of sera from 34 individuals previously identified as seroprotective in the PRN assay.

PPV and NPV are positive and negative predicted values.

## Discussion

The assays most often used to detect measles antibodies are the PRN test and commercial ELISAs. The PRN is a sensitive and reliable assay for measuring measles neutralizing antibodies; however, it is laborious and not easily transferrable between labs. Attempts have been made to streamline the neutralization assay using green fluorescent-tagged MV and counting fluorescent foci or reading total fluorescence per well [14, 15]. However, these assays take 3–7 days to complete. While ELISAs are rapid, they do not detect functional antibodies. Since ELISAs are often designed to have a low false-positive rate, these assays may underestimate the true prevalence of measles immunity within a population. We describe the development of a rapid, high-throughput, specific, and sensitive measles microneutralization assay with a RT-qPCR endpoint. The MN-RT-qPCR assay is robust over a wide range of MV inputs indicating compliance with the Percentage Law. This assay can be used to quickly discriminate between measles susceptible and immune individuals. Additionally, this quantitative assay measures anti-measles neutralizing antibodies across a wide linear range and may provide clues about measles control and recent exposure to wild-type virus even within highly-vaccinated populations. Vaccine recipients typically have GMCs of measles neutralizing antibodies below 5000 mIU/mL while vaccinated individuals recently exposed to wild-type MV may develop concentrations of anti-measles neutralizing antibodies ≥40,000 mIU/mL [16].

The MN-RT-qPCR assay had excellent agreement with results obtained using a traditional PRN assay in identifying human serum samples seronegative for measles neutralizing antibodies compared to those that were seropositive, with sensitivity and specificity ≥95%.

However, estimates of agreement were lower when we compared the ability of the MN-RT-qPCR test to identify serum samples with measles antibodies at or above a seroprotective threshold. A concentration of ≥200 mIU/mL of neutralizing antibodies is considered to be correlated with protection against severe measles disease when measured using a PRN test with an ND_50_ endpoint. Therefore, we first determined the equivalent ND_90_ result using the MN-RT-qPCR test using incurred samples with known ND_50_ results of 200 ± 20 mIU/mL. When these samples were tested in the MN-RT-qPCR assay, the mean GMC was ~300 mIU/mL. When the results of the MN-RT-qPCR assay were analyzed and compared to the ND_50_ results obtained from PRN test using ROC analysis, the threshold for protection was estimated at ~180 mIU/mL. It is reasonable to assume that a higher concentration of antibody is needed to neutralize 90% of the input virus when compared to the concentration needed to neutralize 50%. Therefore, a GMC of ≥300 mIU/mL is a conservative cut-point to use as a preliminary ND_90_ threshold for protection against severe measles disease when testing serum samples using the MN-RT-qPCR test. Furthermore, serum samples with a PRN ND_50_ of ~200 mIU/mL had a calculated PRN ND_90_ value of 278 mIU/mL, providing additional support for a proposed MN-RT-qPCR threshold of 300 mIU/mL to identify individuals likely to be protected against severe measles. When the analysis was conducted looking at the sensitivity, specificity, accuracy, NPV, and PPV of the MN-RT-qPCR vs. PRN assay using the conventional seroprotective cutoff of ≥200 mIU/mL, there was only a slight improvement in the outcome for sensitivity, accuracy and NPV compared to the use of ≥300 mIU/mL as the cutoff. The use of ≥300 mIU/mL as the proposed seroprotective threshold using ND_90_ endpoints obtained using the MN-RT-qPCR assay will need to be explored and confirmed in future epidemiological studies.

Nevertheless, it is encouraging to note that Bland-Altman analyses of the PRN ND_50_ compared to the MN-RT-qPCR ND_90_ assay indicated that the assays were in good agreement and that agreement across assays improved when data from both assays were compared using ND_90_ endpoints. The two-way comparison across assays indicated that there was excellent agreement between the MN-RT-qPCR and PRN assays when identifying those individuals who were seropositive or seronegative for measles neutralizing antibodies. This analysis was conducted using a method with set criteria and cutoffs; if there are any deviations in the test method then the criteria and cutoffs would need to be re-evaluated.

This study has several limitations that should be noted. Firstly, we only tested a small set of samples representing pre- and post-vaccination samples and selected to represent a limited range of measles antibody concentrations from <4 to 700 mIUmL. An assay used in surveillance programs should test a larger number of samples and be able to detect a wide range of neutralizing antibody concentrations from both post-vaccination and post-exposure samples. Secondly, the MN-RT-qPCR assay is based on SYBR green technology. There are inherent sensitivity limitations to this method, especially if the target is in low abundance. In addition, primer dimers and contaminants can skew the Ct value for a sample which may result in an artificially low or high measles antibody concentration. Although we used disassociation curves to confirm the specificity of the amplicon, an oligonucleotide probe-based method may improve the specificity and sensitivity of this assay. The main disadvantage of the MN-RT-qPCR assay is the cost. This assay is more expensive than the PRN test due to reagents needed to harvest cell lysates and amplify the gene target and any additional cost (i.e. oligonucleotide probe) may exponentially escalate when using this assay for sero-surveillance studies. The estimated cost for a single sample (8 dilutions in duplicate) is ~$25.00 compared to ~$8.00 for the PRN assay (6 dilutions in duplicate). The bulk of the cost for the MN-RT-qPCR is the cell lysis buffer; however, the overall cost of the MN-RT-qPCR can be reduced by 50% by substituting an in-house lysis buffer for a commercial buffer [17].

We have shown that the MN-RT-qPCR assay can correctly discriminate between measles seropositive and seronegative sera based on comparison with the PRN test. This assay obeys the Percentage Law and is capable of measuring measles neutralizing antibodies over a broad linear range from 30 to 3000 mIU/mL. The MN-RT-qPCR measles neutralization assay is a simple method and the RT-qPCR aspect of the assay and data analysis are amenable to automation that may prove to be useful in large-scale global measles surveillance programs. Currently, this assay uses the low-passage Edmonston measles virus derived from the same parent virus as several vaccine strains. Since measles virus is a single serotype, the results obtained from neutralization assays using low-passage Edmonston measles virus should be indicative of the ability to neutralize diverse genotypes of measles virus. Nevertheless, there may be subtle antigenic differences between genotypes that might play a role in neutralization. Therefore, we are in the process of adapting this assay to use B3, H1, or D8 measles genotypes as the input virus to answer the question of cross-protective immunity.

## Supporting information

S1 Fig Optimization of annealing temperature of primer sets targeting the N gene of measles virus. Total RNA was purified from Vero cells infected with low-passage Edmonston MV and serially diluted (10-fold) into Vero cell lysate obtained from uninfected Vero cells (10 ng/μL to 0.0001 ng/μL). One microliter of each dilution was subjected to one-step SYBR green RT-qPCR using primer sets diluted at 300 nM and evaluated in a temperature gradient flanking the recommended Tm (as determined by ABI software). The annealing temperature ranges were (A) 55 to 67°C for NPB1/NPB2, (B) 46 to 58°C for N-F/N-R, and (C) 48 to 60°C for N3-F/N3-R primer sets. The mean threshold cycle (Ct, N = 2) is plotted against log_10_ for each RNA standard dilution.
(TIF)

S2 Fig Optimization of primer concentrations targeting the N gene of measles virus. Total RNA was purified from Vero cells infected with low-passage Edmonston MV and serially diluted (10-fold) into Vero cell lysate obtained from uninfected Vero cells (10 ng/μL to 0.0001 ng/μL). One microliter of each dilution was subjected to one-step SYBR green RT-qPCR using primer sets diluted from 100 to 1000nM at annealing temperatures of (A) 67°C for NPB1/NPB2, (B) 57°C for N-F/N-R, and (C) 60°C for N3-F/N3-R primer sets. The mean threshold cycle (Ct, N = 2) is plotted against log_10_ for each RNA standard dilution.
(TIF)

S3 Fig Impact of minimally diluted sample on MV inhibition. Human immune globulin depleted (IgΔ) serum was serially diluted 2-fold in PBS and assessed in MN-RT-qPCR assay as described in Materials and methods. Values represent the mean ± standard deviation (N = 8 replicates).
(TIF)

S4 Fig Correlation between MN-RT-qPCR and PRN. Neutralization assays were performed with low-passage Edmonston MV using a panel of human serum samples (N = 136). The concentrations were log transformed prior to the analysis. The log_10_ MN-RT-qPCR GMC were plotted against the log_10_ PRN GMCs for the individual samples. (A) Analysis of MN-RT-qPCR ND_90_ vs PRN ND_50_. (B) Analysis of MN-RT-qPCR ND_90_ vs. PRN ND_90_ values of measles neutralizing antibodies in milli-International units. The dotted vertical and horizontal lines represent respective cutoff for each assay.
(TIF)

S1 Table Optimization of the incubation time for virus/antibody mixtures. (DOCX)

S2 Table Neutralization activity of rabbit polyclonal antisera against the homologous virus. (DOCX)

## References

[1] Chen RT, Markowitz LE, Albrecht P, Stewart JA, Mofenson LM, Preblud SR, et al Measles antibody: reevaluation of protective titers. The Journal of Infectious Diseases. 1990;162:1036–42. 10.1093/infdis/162.5.1036 2230231

[2] Strebel P, Papania M, D G.H., Halsey NA. Measles Vaccine In: P S., O W., O P., editors. Vaccines. 5th ed: Saunders Elsevier; 2008 p. 353–98.

[3] Dabbagh A, Laws RL, Steulet C, Dumolard L, Mulders MN, Kretsinger K, et al Progress Toward Regional Measles Elimination—Worldwide, 2000–2017. MMWR Morb Mortal Wkly Rep. 2018;67(47):1323–9. Epub 2018/11/30. 10.15585/mmwr.mm6747a6 potential conflicts of interest. No potential conflicts of interest were disclosed. 30496160 PMC6276384

[4] World Health Organization. Global Vaccine Action Plan 2011–2020 [cited 2018 8 November]. http://www.who.int/immunization/global_vaccine_action_plan/GVAP_doc_2011_2020/en/.

[5] Varada JC, Teferedegne B, Crim RL, Mdluli T, Audet S, Peden K, et al A neutralization assay for respiratory syncytial virus using a quantitative PCR-based endpoint assessment. Virol J. 2013;10:195 Epub 2013/06/19. 10.1186/1743-422X-10-195 23767960 PMC3686610

[6] Hosken N, Plikaytis B, Trujillo C, Mahmood K, Higgins D, Participating Laboratories Working G. A multi-laboratory study of diverse RSV neutralization assays indicates feasibility for harmonization with an international standard. Vaccine. 2017;35(23):3082–8. 10.1016/j.vaccine.2017.04.053 28476625 PMC5439532

[7] Hummel KB, Lowe L, Bellini WJ, Rota PA. Development of quantitative gene-specific real-time RT-PCR assays for the detection of measles virus in clinical specimens. J Virol Methods. 2006;132(1–2):166–73. 10.1016/j.jviromet.2005.10.006 . 16274752

[8] Nakayama T, Mori T, Yamaguchi S, Sonoda S, Asamura S, Yamashita R, et al Detection of measles virus genome directly from clinical samples by reverse transcriptase-polymerase chain reaction and genetic variability. Virus Res. 1995;35(1):1–16. . 7754670 10.1016/0168-1702(94)00074-m

[9] Plumet S, Gerlier D. Optimized SYBR green real-time PCR assay to quantify the absolute copy number of measles virus RNAs using gene specific primers. J Virol Methods. 2005;128(1–2):79–87. 10.1016/j.jviromet.2005.03.020 . 15913798

[10] Cohen BJ, Audet S, Andrews N, Beeler J. Plaque reduction neutralization test for measles antibodies: Description of a standardised laboratory method for use in immunogenicity studies of aerosol vaccination. Vaccine. 2007;26(1):59–66. 10.1016/j.vaccine.2007.10.046 18063236

[11] Klasse PJ, Sattentau QJ. Occupancy and mechanism in antibody-mediated neutralization of animal viruses. J Gen Virol. 2002;83(Pt 9):2091–108. 10.1099/0022-1317-83-9-2091 . 12185262

[12] Andrewes CH, Elford WJ. Observations on Anti-Phage Sera. I: “The Percentage Law”. The British Journal of Experimental Pathology. 1933;14(6):367–76.

[13] Albrecht P, Herrman K, Burns GR. Role of virus strain in conventional and enhance measles plaque neutralization test. Journal of Virological Methods. 1981;3:251–60. 7334066 10.1016/0166-0934(81)90062-8

[14] Fujino M, Yoshida N, Kimura K, Zhou J, Motegi Y, Komase K, et al Development of a new neutralization test for measles virus. J Virol Methods. 2007;142(1–2):15–20. 10.1016/j.jviromet.2007.01.001 17320979

[15] Haralambieva IH, Ovsyannikova IG, Vierkant RA, Poland GA. Development of a novel efficient fluorescence-based plaque reduction microneutralization assay for measles virus immunity. Clin Vaccine Immunol. 2008;15(7):1054–9. 10.1128/CVI.00008-08 18463223 PMC2446644

[16] Sowers SB, Rota JS, Hickman CJ, Mercader S, Redd S, McNall RJ, et al High Concentrations of Measles Neutralizing Antibodies and High-Avidity Measles IgG Accurately Identify Measles Reinfection Cases. Clin Vaccine Immunol. 2016;23(8):707–16. Epub 2016/06/24. 10.1128/CVI.00268-16 . 27335386 PMC4979181

[17] Shatzkes K, Teferedegne B, Murata H. A simple, inexpensive method for preparing cell lysates suitable for downstream reverse transcription quantitative PCR. Sci Rep. 2014;4:4659 10.1038/srep04659 24722424 PMC3983595

