# Correction: Development of a high-throughput assay to measure measles neutralizing antibodies

**DOI:** 10.1371/journal.pone.0223156

**Published:** 2019-09-23

**Authors:** The PLOS ONE Staff

In Table 1, the forward and reverse primer sequences for each primer set were merged into one line. The forward and reverse primer sequences for each primer should be on their own line. Please see the correct Table 1 here. The publisher apologizes for the error.

**Table 1:** Primer pairs targeting the N gene of measles virus.

Primer	Sequence	Amplicon size
NPB1	GATCCGCAGGACAGTCGAAGGTC	169
NPB2	AGGGTAGGCGGATGTTGTTCT	
N-F	AGTGAGAATGAGCTACCG	151
N-R	TGTCTAGGGGTGTGCC	
N3-F	TGGCATCTGAACTCGGTATCAC	75
N3-R	TGTCCTCAGTAGTATGCATTGCAA	
