# Supplementary figures and images for: Development of a high-throughput assay to measure measles neutralizing antibodies
Source: PLoS One. 2019 Aug 15;14(8):e0220780. doi: 10.1371/journal.pone.0220780 (PMC6695214; doi:10.1371/journal.pone.0220780)

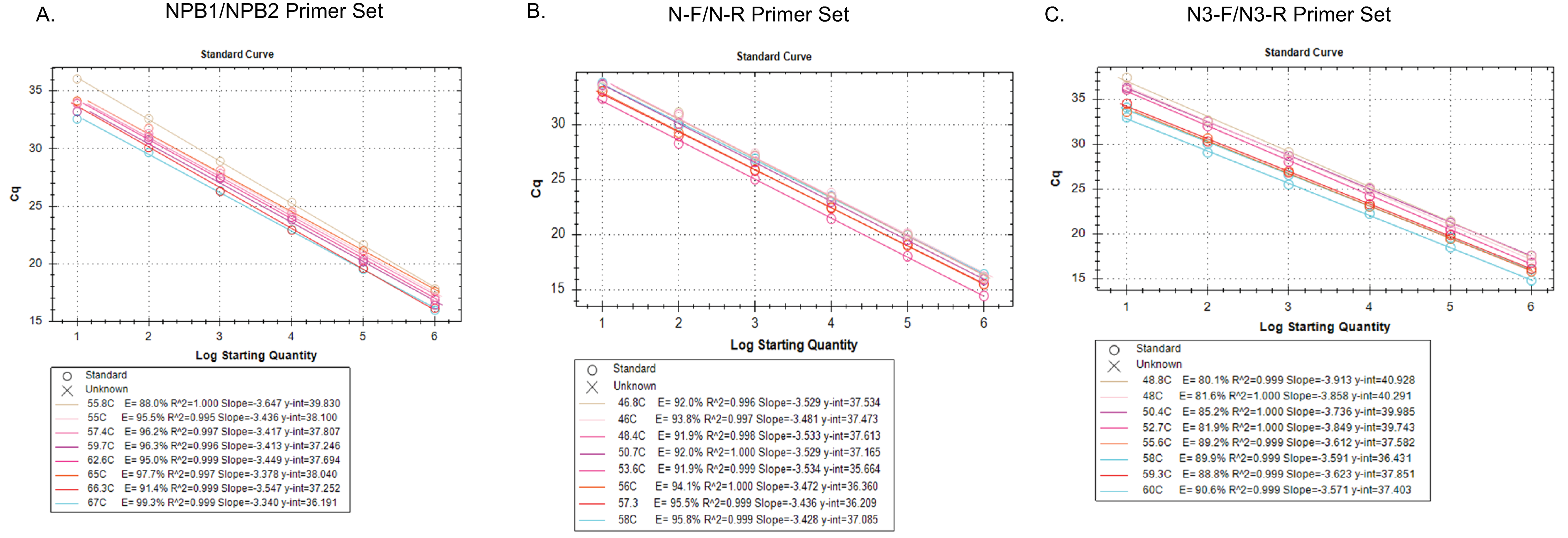

Supplement: S1 Fig — Total RNA was purified from Vero cells infected with low-passage Edmonston MV and serially diluted (10-fold) into Vero cell lysate obtained from uninfected Vero cells (10 ng/μL to 0.0001 ng/μL). One microliter of each dilution was subjected to one-step SYBR green RT-qPCR using primer sets diluted at 300 nM and evaluated in a temperature gradient flanking the recommended Tm (as determined by ABI software). The annealing temperature ranges were (A) 55 to 67°C for NPB1/NPB2, (B) 46 to 58°C for N-F/N-R, and (C) 48 to 60°C for N3-F/N3-R primer sets. The mean threshold cycle (Ct, N = 2) is plotted against log10 for each RNA standard dilution. (TIF) [file pone.0220780.s001.tif]

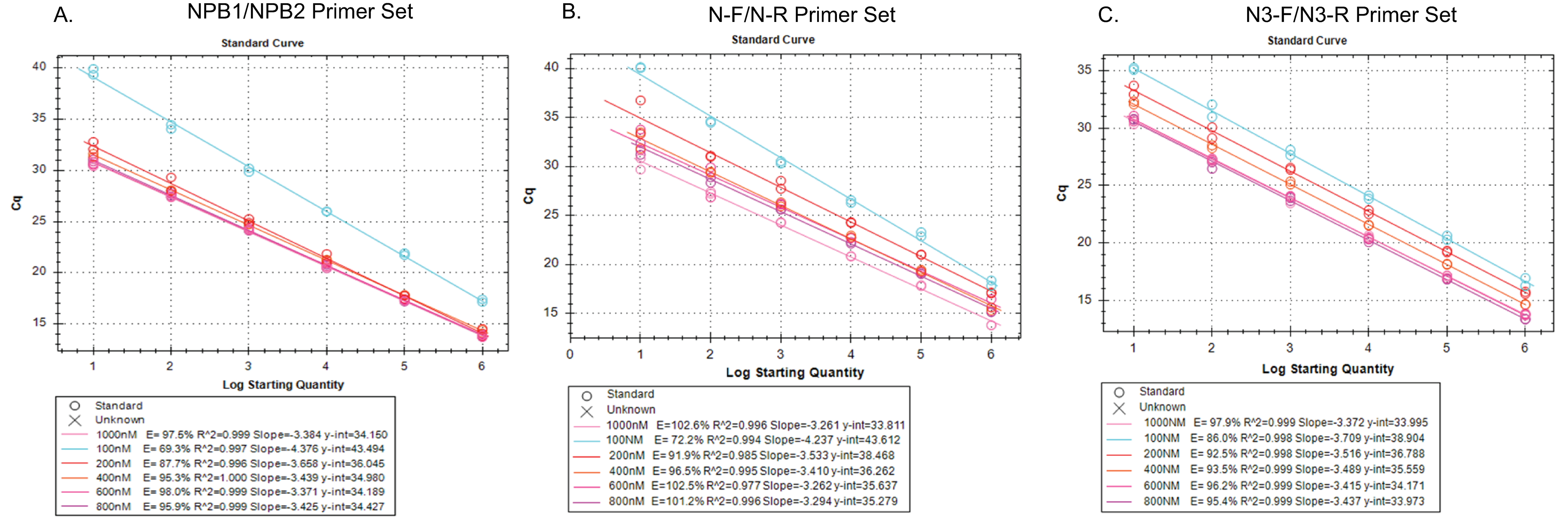

Supplement: S2 Fig — Total RNA was purified from Vero cells infected with low-passage Edmonston MV and serially diluted (10-fold) into Vero cell lysate obtained from uninfected Vero cells (10 ng/μL to 0.0001 ng/μL). One microliter of each dilution was subjected to one-step SYBR green RT-qPCR using primer sets diluted from 100 to 1000nM at annealing temperatures of (A) 67°C for NPB1/NPB2, (B) 57°C for N-F/N-R, and (C) 60°C for N3-F/N3-R primer sets. The mean threshold cycle (Ct, N = 2) is plotted against log10 for each RNA standard dilution. (TIF) [file pone.0220780.s002.tif]

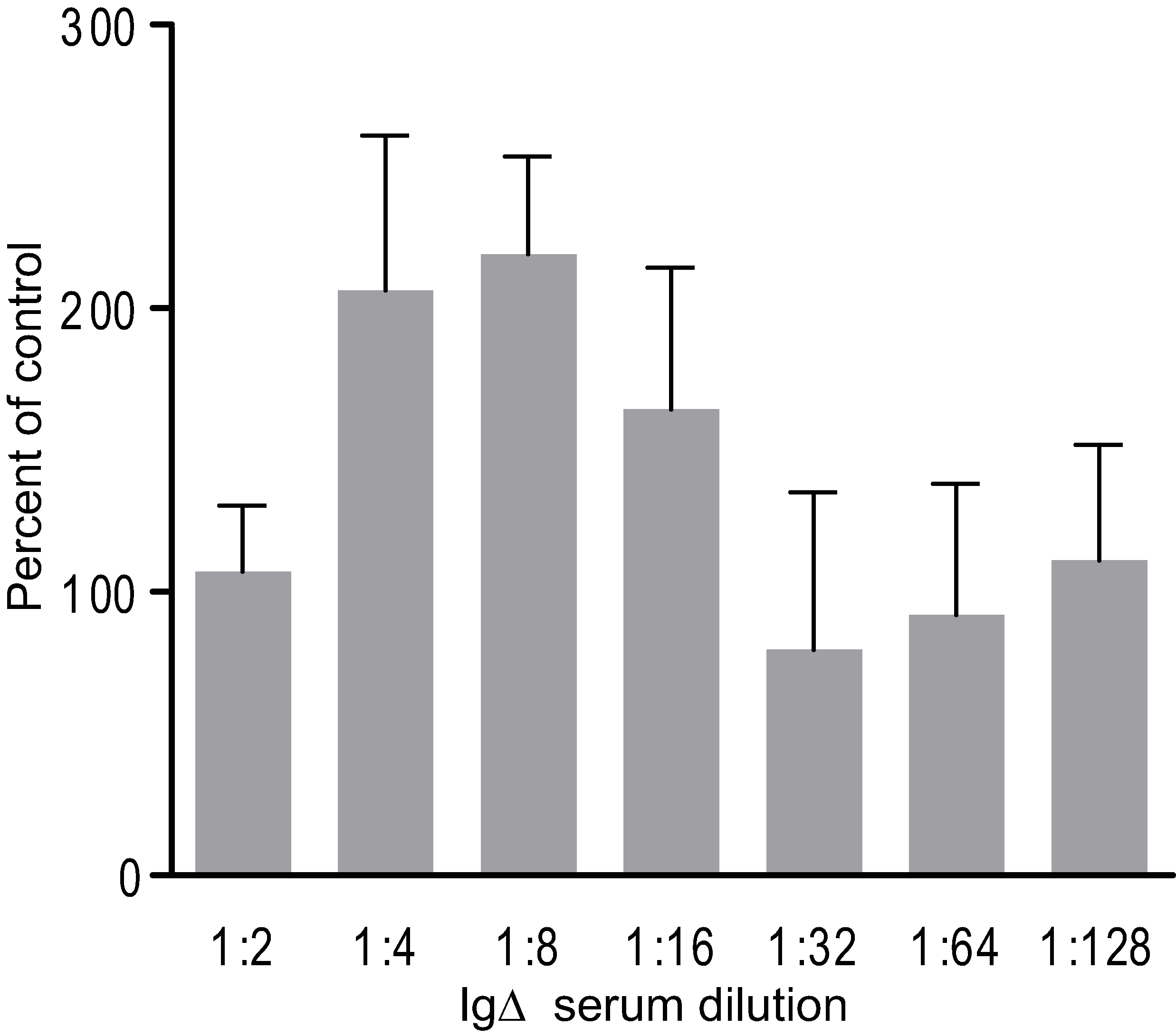

Supplement: S3 Fig — Human immune globulin depleted (IgΔ) serum was serially diluted 2-fold in PBS and assessed in MN-RT-qPCR assay as described in Materials and methods. Values represent the mean ± standard deviation (N = 8 replicates). (TIF) [file pone.0220780.s003.tif]

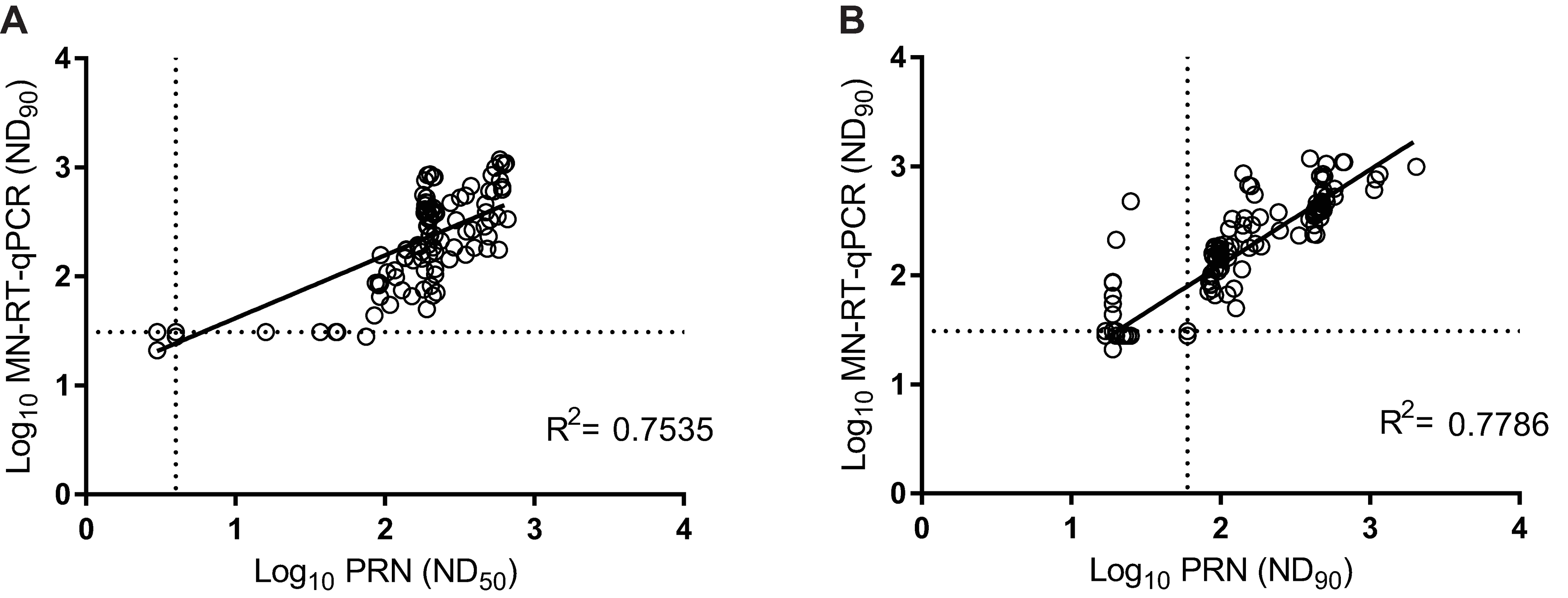

Supplement: S4 Fig — Neutralization assays were performed with low-passage Edmonston MV using a panel of human serum samples (N = 136). The concentrations were log transformed prior to the analysis. The log10 MN-RT-qPCR GMC were plotted against the log10 PRN GMCs for the individual samples. (A) Analysis of MN-RT-qPCR ND90 vs PRN ND50. (B) Analysis of MN-RT-qPCR ND90 vs. PRN ND90 values of measles neutralizing antibodies in milli-International units. The dotted vertical and horizontal lines represent respective cutoff for each assay. (TIF) [file pone.0220780.s004.tif]
