# Supplementary material for: Development of a high-throughput assay to measure measles neutralizing antibodies
Source: PLoS One. 2019 Aug 15;14(8):e0220780. doi: 10.1371/journal.pone.0220780 (PMC6695214; doi:10.1371/journal.pone.0220780)
Supplement: S1 Table — (DOCX) [file pone.0220780.s005.docx]

| S1 Table. Optimization of the incubation time virus/antibody mixtures | | |
| --- | --- | --- |
| Sample | ND_90_ GMC (mIU/mL) | |
|  | 1 hour | 2 hours |
| WHO 3^a^ | 294 | 490 |
| huIgG^b^ | 171 | 277 |
| ^a^ 3^rd^ International WHO Serum Standard 97/648  ^b^ Human reference immune globulin (BEI# 21973) | | |
