# Supplementary material for: Development of a high-throughput assay to measure measles neutralizing antibodies
Source: PLoS One. 2019 Aug 15;14(8):e0220780. doi: 10.1371/journal.pone.0220780 (PMC6695214; doi:10.1371/journal.pone.0220780)
Supplement: S2 Table — (DOCX) [file pone.0220780.s006.docx]

**S2 Table. Neutralization activity of rabbit polyclonal antisera against the homologous virus**

| Sample | Measles GMC (mIU/mL)^a^ |
| --- | --- |
| Rabbit anti-Canarypox Measles-F | 74 |
| Rabbit anti-Canarypox Measles-HA | 4187 |
| Rabbit anti-UV-inactivated-Edmonston measles wild-type | 144 |
| Rabbit anti-Moraten measles vaccine virus | 7242 |
| Rabbit anti-UV-inactivated-Moraten measles vaccine virus | >8200 |
|  |  |
| Sample | RSV GMT^b^ |
| Rabbit anti-UV-inactivated-RSV A2 | 5731 |
| Rabbit anti-UV-inactivated-RSV B1 | 55 |
|  |  |
| Sample | hMPV GMT^c^ |
| Rabbit anti-UV-inactivated-hMPV | 189 |

^a^Measles neutralizing antibody concentrations were determined by PRN as described in Materials and Methods.

^b^The 50% RSV neutralizing antibody titers were determined by PRN assay using RSV-A2 virus as described previously [5].

^c^The 50% hMPV neutralizing antibody titers were determined by in-house fluorescent based microneutralization assay using a hMPV-GFP virus [unpublished method].
